# Entropy Generation Assessment for Wall-Bounded Turbulent Shear Flows Based on Reynolds Analogy Assumptions

**DOI:** 10.3390/e21121157

**Published:** 2019-11-26

**Authors:** Matthias Ziefuss, Nader Karimi, Florian Ries, Amsini Sadiki, Amirfarhang Mehdizadeh

**Affiliations:** 1 Civil and Mechanical Engineering Department, School of Computing and Engineering, University of Missouri-Kansas City, Kansas City, MO 64110, USA; matthias.ziefuss@mail.umkc.edu (M.Z.); mehdizadeha@umkc.edu (A.M.); 2 School of Engineering, University of Glasgow, Glasgow G12 8QQ, UK; Nader.Karimi@glasgow.ac.uk; 3 Department of Mechanical Engineering, Institute of Energy and Power Plant Technology, Technische Universität Darmstadt, 64289 Darmstadt, Germany; Sadiki@ekt.tu-darmstadt.de

**Keywords:** Reynolds Analogy, entropy generation, steady/unsteady calculations

## Abstract

Heat transfer modeling plays a major role in design and optimization of modern and efficient thermal-fluid systems. Further, turbulent flows are thermodynamic processes, and thus, the second law of thermodynamics can be used for critical evaluations of such heat transfer models. However, currently available heat transfer models suffer from a fundamental shortcoming: their development is based on the general notion that accurate prediction of the flow field will guarantee an appropriate prediction of the thermal field, known as the. In this work, an assessment of the capability of the in predicting turbulent heat transfer when applied to shear flows of fluids of different Prandtl numbers will be given. Towards this, a detailed analysis of the predictive capabilities of the concerning entropy generation is presented for steady and unsteady state simulations. It turns out that the provides acceptable results only for mean entropy generation, while fails to predict entropy generation at small/sub-grid scales.

## 1. Introduction

There are various systems where turbulent heat transfer plays an important role in development and optimization. These include cooling systems for nuclear power plants, where liquid metal is used as coolant [1,2]; boiler systems for biomass combustion [3]; and heat exchange devices in petroleum industry [4], to name just a few. Further, all of these systems share important commonalities: first, experimental investigations are either not possible or prohibitively expensive [5], and second, the underlying thermodynamics process must be as efficient as possible to avoid loss of energy. Optimizing these systems requires a detailed insight into the complex dynamics of heat and mass transfer, demanding advanced and reliable models. In addition, various systems employ working fluids with significantly different Prandtl (denoted as Pr) numbers (ranging from Pr≪1 for liquid metal to a few hundreds for crude oils). The variety in Pr numbers stresses the prediction capabilities of turbulent heat transfer models. Further, the challenge of modeling turbulent heat transfer arises from its strong and complex coupling to the turbulent field. Thus, a reliable model for the flow field (momentum transport) is a mandatory condition for a model of turbulent heat transfer. As a consequence, the main focus in research/modeling was on the modeling of momentum field in the past decades [6].

Turbulent momentum and heat transfer are based on the same underlying physical mechanism of cross-streamwise mixing of fluid elements [6]. Thus, the fundamental assumption that a correct prediction of the momentum transport leads to appropriate prediction of the heat transfer is often made. This analogy is based on the eddy diffusivity approach and is well known as Reynolds Analogy [6]. While this concept is a drastic simplification, it is still widely applied to a majority of industrial applications of Computational Fluid Dynamics (CFD) when first-order statistical quantities such as mean temperature and Nußelt number are of interest.

Furthermore, turbulent flows are thermodynamic processes and the directions of all such processes are restricted by the second law of thermodynamics. Thus, this law can be used for critical evaluation of turbulence and heat transfer models as discussed in Reference [7]. In applications, irreversibilities—described by the second law of thermodynamics—decrease the available energy of the working fluid [8]. This leads to an increase of system entropy and entropy generation [9,10,11]. In conjunction with heat transfer and fluid mechanic principles, it is possible to evaluate the impact of irreversibilities related to heat transport and thermo-fluid systems. Various investigations using the entropy concept including different configurations and physical processes with a variety of numerical and analytical approaches to better understanding the process can be found in References [7,12,13,14,15].

Based on this concept, only a few Direct Numerical Simulation (DNS) can be found in the literature [16,17,18,19,20,21], which are restricted to simple geometries and low-to-medium Reynolds numbers due to the high computational cost. To overcome this problem, Reynolds Averaged Navier Stokes equation (RANS) approaches have been often used to study entropy generation dynamics at high Reynolds numbers. These investigations are reported in few studies [22,23,24,25,26]. However, it is well known that prediction capabilities of RANS models are limited when dealing with turbulent flows with large scale and unsteady characteristics. Unsteady approaches could offer a potential alternative strategy that allow prediction of unsteady dynamics of the flow field, such as hybrid Unsteady Reynolds Averaged Navier Stokes equation (URANS)/Large Eddy Simulation (LES). These overcome restrictions by DNS and RANS simulations to predict flow and thermal statistics accurately yet computationally affordable. Despite the potential of LES and hybrid approaches, only a few publications using these concepts for entropy analyses are available [7,27].

Concerning heat transfer modeling, it is worth noting that both RANS (steady) and unsteady approaches employ mainly the to predict the thermal quantities (total/sub-grid part). This investigation aims to provide a comprehensive assessment of capabilities of the to predict the entropy generation dynamics particularly through heat transfer in different turbulent environments (working fluids with different Prandtl numbers).

The rest of this paper is organized as follows: In Section 2, the employed turbulence models along with relevant transport equations will be presented and discussed. In Section 3, an overview of test cases and numerical approach is provided. Results obtained from the simulations are presented and discussed in Section 4. The paper ends up with a summary and conclusion in Section 5.

## 2. Governing Equations

The current study aims to provide a comprehensive assessment of the prediction capabilities of the for entropy production when applied to turbulent, attached, wall-bounded shear flows of fluids with different Pr numbers. Towards this end, various aspects of this analogy regarding entropy production will be investigated. First, the sensitivity of the with respect to the turbulence model employed to predict the flow field will be investigated. Therefore, two different turbulence models, i.e., k−ω−SST (Shear Stress Transport) and k−ϵ−ζ−f, will be used for steady state (RANS) simulations. In order to prelude effects of numerical instabilities/uncertainties on the model performance, 3-dimensional domains with appropriate mesh resolutions have been used for the RANS simulations.

As the next step, performance of the Reynolds Analogy in unsteady simulations where the analogy operates as a sub-grid-scale (sgs) model is assessed. Sensitivity to grid resolution is investigated as it is an indicator of basic properties of sgs models. The k−ω−SST-IDDES (Improved Delayed Detached Eddy Simulation) model will be used for the unsteady simulations. This model is a hybrid URANS/LES model and is able to provide an accurate prediction of the flow field—comparable to wall-modeled LES—at affordable computational cost [28]. The mathematical formulation of turbulence models used in the current study in conjunction with other transport equations (energy, temperature variance, and entropy) will be presented and discussed in the following.

### 2.1. Turbulence Models

#### 2.1.1. The k−ω−SST Model

The k−ω−SST model is one of the most commonly used models. It employs two transport equations, one for turbulent kinetic energy *k* and one for the inverse of dissipation rate ω to provide necessary turbulence scales [29]. The model equations read as follows:

(1) $$\frac{Dk}{Dt}=\frac{\partial }{\partial x_{i}}\left[\left(\nu +\frac{\nu_{t}}{\sigma_{k}}\right)\frac{\partial k}{\partial x_{i}}\right]+\tilde{P_{k}}-\beta^{☆}\rho \omega k,$$

(2) $$\frac{D\omega }{Dt}=\frac{\partial }{\partial x_{i}}\left[\left(\nu +\frac{\nu_{t}}{\sigma_{\omega }}\right)\frac{\partial \omega }{\partial x_{i}}\right]+2\left(1-F_{1}\right)\frac{\sigma_{\omega 2}}{\omega }\frac{\partial k}{\partial x_{i}}\frac{\partial \omega }{\partial x_{i}}+\frac{\gamma }{\nu_{t}}P_{k}-\beta \omega^{2},$$

with $\tilde{P_{k}}=\min\left(P_{k};c_{l}\epsilon \right)$ and $P_{k}=\tau_{ij}\frac{\partial \bar{u_{i}}}{\partial x_{j}}$ as mechanical turbulent production. Further details on model constants and functions, i.e., $c_{l},\beta ,\beta^{☆},\gamma ,\tau_{ij},\sigma_{\omega 2}$, and $F_{1}$, are provided in Reference [29].

#### 2.1.2. The k−ϵ−ζ−f Model

The k−ϵ−ζ−f is well known to be able to predict near-wall effects in shear flows [30]. In addition to the transport equation for kinetic energy *k* and its dissipation ε, two more equations are solved. The first one is a transport equation for the velocity scale ratio $\zeta =\bar{v^{2}}/k$ and the second one is an elliptic relaxation concept, *f*, to sensitize ζ. For brevity, the model is referred as the ζ−f model. The model equations are as follows:

(3) $$\frac{Dk}{Dt}=\frac{\partial }{\partial x_{i}}\left[\left(\nu +\frac{\nu_{t}}{\sigma_{k}}\right)\frac{\partial k}{\partial x_{i}}\right]+P_{k}-\varepsilon ,$$

(4) $$\frac{D\varepsilon }{Dt}=\frac{\partial }{\partial x_{i}}\left[\left(\nu +\frac{\nu_{t}}{\sigma_{\varepsilon }}\right)\frac{\partial \varepsilon }{\partial x_{i}}\right]+\frac{C_{\varepsilon 1}P_{k}-C_{\varepsilon 2}\varepsilon }{\tau },$$

(5) $$\frac{D\zeta }{Dt}=\frac{\partial }{\partial x_{i}}\left[\left(\nu +\frac{\nu_{t}}{\sigma_{\zeta }}\right)\frac{\partial \zeta }{\partial x_{i}}\right]-\frac{\zeta }{k}P_{k}+f,$$

(6) $$L^{2}\frac{\partial^{2}f}{\partial x_{i}^{2}}-f=\frac{1}{\tau }\left(C_{1}+C_{2}^{{}^{\prime }}\frac{P_{k}}{\varepsilon }\right)\left(\zeta -\frac{2}{3}\right),$$

with $P_{k}=\tau_{ij}\frac{\partial \bar{u_{i}}}{\partial x_{j}}$ and $\nu_{t}=C_{\mu }\zeta k\tau$. Further details on model constants and functions, i.e., $\tau_{ij},\tau ,C_{1},$ $C_{2}^{\prime },C_{\epsilon 1}$, and $C_{\epsilon 2}$, are provided in Reference [30].

#### 2.1.3. The k−ω−SST-IDDES Model

k−ω−SST-IDDES employs a modified version of the k−ω−SST model to improve near-wall prediction and to enable unsteady calculations. It is defined with two transport equations for *k* and ω:

(7) $$\frac{Dk}{Dt}=\frac{\partial }{\partial x_{i}}\left[\left(\nu +\frac{\nu_{t}}{\sigma_{k}}\right)\frac{\partial k}{\partial x_{i}}\right]+P_{k}-\sqrt{k^{3}}/l_{IDDES},$$

(8) $$\frac{D\omega }{Dt}=\frac{\partial }{\partial x_{i}}\left[\left(\nu +\frac{\nu_{t}}{\sigma_{\omega }}\right)\frac{\partial \omega }{\partial x_{i}}\right]+2\left(1-F_{1}\right)\frac{\sigma_{\omega 2}}{\omega }\frac{\partial k}{\partial x_{i}}\frac{\partial \omega }{\partial x_{i}}+\frac{\gamma }{\nu_{t}}P_{k}-\beta \omega^{2},$$

where blending function $F_{1}$; production term $P_{k}$; and model constants γ, $\sigma_{k}$, $\sigma_{w}$, $\sigma_{w2}$, and β are imported from the original k−ω−SST model [29]. It should be noted that, within k−ω−SST-IDDES, only the destruction term in the *k*-equation is modified by introducing the $l_{IDDES}$ term, whereas the ω equation remains unchanged. $l_{IDDES}$ is responsible for triggering a transition from URANS mode into a scale-resolving mode. A detailed description of this methodology can be found in References [31,32].

### 2.2. Energy Equation and Heat Transfer Model

The Reynolds-averaged energy equation follows [33]:

(9) $$\rho c_{p}\frac{DT}{Dt}=S_{T}+\frac{\partial }{\partial x_{i}}\left[\left(\lambda \frac{\partial T}{\partial x_{i}}\right)-\rho c_{p}\bar{\theta u_{i}}\right],$$

Assuming incompressible flow and constant physical properties and neglecting addition source, $S_{T}$, and terms such as radiation, the equation for the mean temperature *T* can be written as below:

(10) $$\frac{DT}{Dt}=\frac{\partial }{\partial x_{i}}\left[\left(\frac{\nu }{Pr}\frac{\partial T}{\partial x_{i}}\right)-\bar{\theta u_{i}}\right].$$

The quantity $\bar{\theta u_{i}}$ on the right-hand side is called turbulent heat flux and is the Reynolds-averaged fluctuating velocity–temperature correlation. This quantity needs to be modeled in order to close the equation.

**Figure 1:**
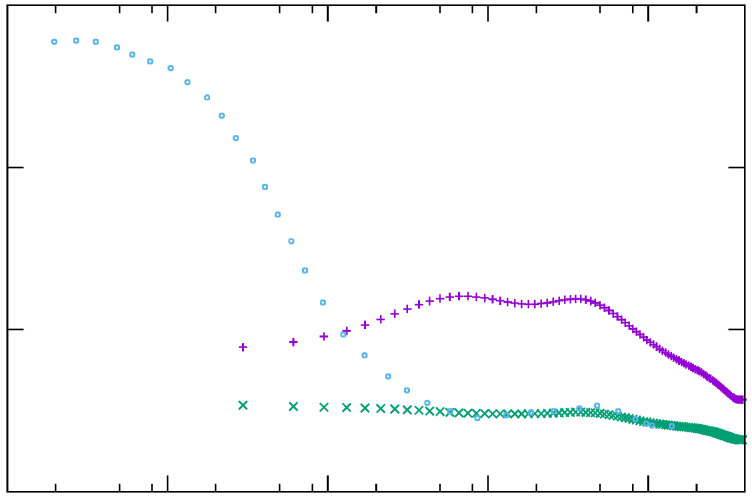
Variation of turbulent Prandtl number $\sigma_{t}$ at different ${Re}^{\tau }$ and Pr numbers: ${Re}^{\tau }=395$ with Pr=0.025 [35] (+) and Pr=0.71 [35] (×), and ${Re}^{\tau }=150$ with Pr=200 [36] (⚬).

The simplest and mostly used approach to model the turbulent heat flux is the. This approach is based on the assumption that the momentum and thermal layer overlay and, thus, have the same thickness. Therefore, it is assumed that an accurate computation of the momentum transport leads to an accurate prediction of the temperature field. In addition, it is assumed that the turbulent heat flux is proportional to the mean temperature gradient [34], which leads to the following relation:

(11) $$\bar{\theta u_{i}}=-\frac{\nu_{t}}{\sigma_{t}}\frac{\partial T}{\partial x_{i}},$$

with $\sigma_{t}$ as the turbulent Prandtl number, usually taken constant and equal to 0.9 [6]. This value is suitable/appropriate only for fluids with Pr numbers around unity. Concerning low number fluids, this value is significantly lower than the averaged reference value obtained from DNS; see Figure 1. However, this value provides a reasonable estimation for high Pr number fluids except for regions very close to solid surfaces, i.e., $y^{+}<3$; see Figure 1.

Moreover, it is immediately clear that the capability of the is limited to only first-order statistics in nonhomogeneous directions and, thus, fails to predict the heat flux in the homogeneous direction when employed for steady-state simulations.

In case of unsteady calculations, the internal energy equation (Equation (10)) as well as the (Equation (11)) take the following form:

(12) $$\begin{array}{c} \frac{DT}{Dt}=\frac{\partial }{\partial x_{i}}\left[\left(\frac{\nu }{Pr}\frac{\partial T}{\partial x_{i}}\right)-{\bar{\theta u_{i}}}^{sgs}\right]\;\mathrm{and}\;=-\frac{{\nu_{t}}^{sgs}}{\sigma_{t}^{sgs}}\frac{\partial T}{\partial x_{i}}, \end{array}$$

where and ${\bar{\theta u_{i}}}^{sgs}$ and ${\nu_{t}}^{sgs}$ represent sub-grid heat flux and sub-grid eddy viscosity, respectively. Thus, the total heat flux is the sum out of sub-grid-scale (sgs) and resolved (res) components.

### 2.3. Temperature Variance Equation

As for a turbulent flow field, for which the characteristic time is provided by $\tau_{m}=k/\varepsilon$, it is also of interest to introduce a characteristic time scale for thermal mixing, which can be given as $\tau_{\theta }=/{2\varepsilon }_{\theta }$, where $\bar{\theta^{2}}$ is the temperature variance and is its dissipation. These quantities are important for entropy analyses, as will be shown later. The modeled transport equation for $\bar{\theta^{2}}$ reads as follows [6]:

(13) $$\frac{D\bar{\theta^{2}}}{Dt}=2P_{\bar{\theta^{2}}}-2\varepsilon_{\theta }+\frac{\partial }{\partial x_{i}}\left[\left(\frac{\nu }{Pr}+\frac{\nu_{t}}{\sigma_{k}}\right)\frac{\partial \bar{\theta^{2}}}{\partial x_{i}}\right],$$

where $P_{\bar{\theta^{2}}}=-\bar{\theta u_{i}}\partial T/\partial x_{i}$ is the production of temperature variance and $\varepsilon_{\theta }$ is the dissipation of temperature variance. Introducing an additional transport equation for this quantity would be the most consistent approach to close Equation (13). However, closing this equation is more complex compared to modeling the equation for the dissipation of turbulent kinetic energy ε. As stated in Reference [6], twice as many free parameters, including two turbulent time scales (mechanical and thermal), and two production terms need to be determined. These issues have been discussed in a few investigations [37,38,39,40].

However, often, a simpler approach that assumes a constant thermal to mechanical time-scale ratio, denoted as $R=\tau_{\theta }/\tau_{m}$, is used to provide information on the thermal time scale [6,41]. Several studies [34,42,43] have shown that the assumption of a constant ratio—with a typical value of R=0.5—works pretty well for fluids with Pr number around unity. Nevertheless, it is commonly used even when dealing with Pr numbers significantly different than unity [6], despite the lack of extensive assessment and validation. Using the typical value of 0.5 for R leads to the following relation for $\varepsilon_{\theta }$ and is used in this study along with the Reynolds Analogy to determine $\varepsilon_{\theta }$ in the temperature variance equation:

(14) $$\varepsilon_{\theta }=\varepsilon \frac{\bar{\theta^{2}}}{k}.$$

In case of unsteady calculations, the transport equation for the temperature variance (Equation (13)) takes the following form:

(15) $$\frac{D{\bar{\theta^{2}}}^{sgs}}{Dt}=2P_{sgs}^{\theta^{2}}-2\varepsilon_{\theta }^{sgs}+\frac{\partial }{\partial x_{i}}\left[\left(\frac{\nu }{Pr}+\frac{\nu_{t}^{sgs}}{\sigma_{k}^{sgs}}\right)\frac{\partial {\bar{\theta^{2}}}^{sgs}}{\partial x_{i}}\right],$$

with

(16) $$P_{sgs}^{\theta^{2}}=-{\bar{\theta u_{i}}}^{sgs}\frac{\partial T}{\partial x_{i}}\;\mathrm{and}\;\varepsilon_{\theta }^{sgs}=\frac{\varepsilon^{sgs}{\bar{\theta^{2}}}^{sgs}}{k^{sgs}}.$$

Thus, the total temperature variance is the sum of res and sgs components.

### 2.4. Entropy Equation

Entropy generation due to different mechanisms will be presented and discussed in the following. Under the assumptions of Cartesian coordinates, incompressible fluid, single-phase flow, and non-reacting and Fourier heat conduction, the second law of thermodynamics can be expressed as a local imbalance as below [44]:

(17) $$\rho \frac{Ds}{Dt}+\frac{\partial }{\partial x_{i}}\left[\frac{q_{i}}{\Theta }\right]=\Pi_{v}+\Pi_{q}\geq 0.$$

The two production terms, $\Pi_{v}$ and $\Pi_{q}$, represent important mechanisms for entropy production. If production due to radiation is neglected, these two are as below:

(18) $$\Pi_{v}=\frac{\mu }{\Theta }\left(\frac{\partial U_{i}}{\partial x_{j}}+\frac{\partial U_{j}}{\partial x_{i}}\right)\frac{\partial U_{i}}{\partial x_{j}},$$

(19) $$\Pi_{q}=\frac{1}{\Theta^{2}}q_{i}\frac{\partial \Theta }{\partial x_{i}}=\frac{\lambda }{\Theta^{2}}\frac{\partial \Theta }{\partial x_{i}}\frac{\partial \Theta }{\partial x_{i}},$$

where $\Pi_{v}$ is the production due to the viscous dissipation and $\Pi_{q}$ is the production by heat transfer due to finite temperature gradients. These terms are always positive and, thus, act as source terms. Both terms need to be calculated for entropy generation analysis, since they are responsible for irreversibilities evolving in heat transferring viscous fluid flows.

#### 2.4.1. Entropy Production—Steady-State Calculations

In the concept of Reynolds Averaged Navier Stokes equation (RANS), Equation (17) holds the instantaneous values, and following the Reynolds decomposition [45,46], this equation can be decomposed into mean and fluctuating parts. Accordingly, entropy production due to viscous dissipation can be decomposed into mean and fluctuating parts, i.e., $\Pi_{v}={\bar{\Pi }}_{v}+\Pi_{v}^{{}^{\prime }}$, with

(20) $${\bar{\Pi }}_{v}=\frac{\mu }{T}\left(\frac{\partial {\bar{u}}_{i}}{\partial x_{j}}+\frac{\partial {\bar{u}}_{j}}{\partial x_{i}}\right)\frac{\partial {\bar{u}}_{i}}{\partial x_{j}},$$

(21) $$\Pi_{v}^{{}^{\prime }}=\frac{\mu }{T}\bar{(\frac{\partial u_{i}}{\partial x_{j}}+\frac{\partial u_{j}}{\partial x_{i}})\frac{\partial u_{i}}{\partial x_{j}}}=\frac{\mu }{T}\underset{A}{\underset{︸}{\bar{{\left(\frac{\partial u_{i}}{\partial x_{j}}\right)}^{2}}}}.$$

Calculation of ${\bar{\Pi }}_{v}$ is possible using knowledge on mean values of velocity and temperature, which are always known in calculations. In contrast, $\Pi_{v}^{\prime }$ is not closed and has to be modeled. Considering the exact equation for turbulent dissipation, $\varepsilon =\nu \bar{{\left(\partial u_{i}/\partial x_{j}\right)}^{2}}$, and thus by assuming an equivalence between ε and the term *A*, discussed in Reference [45], Equation (21) can be approximated via known mean values as below:

(22) $$\Pi_{v}^{\prime }=\frac{\rho }{T}\varepsilon .$$

Similarly, entropy production due to heat transfer can be decomposed into mean and fluctuation parts, i.e., $\Pi_{q}={\bar{\Pi }}_{q}+\Pi_{q}^{\prime }$, with

(23) $${\bar{\Pi }}_{q}=\frac{\lambda }{T^{2}}\frac{\partial T}{\partial x_{i}}\frac{\partial T}{\partial x_{i}},$$

(24) $$\Pi_{q}^{\prime }=\frac{\lambda }{T^{2}}\bar{\frac{\partial \theta }{\partial x_{i}}\frac{\partial \theta }{\partial x_{i}}}=\frac{\lambda }{T^{2}}\underset{B}{\underset{︸}{\bar{{\left(\frac{\partial \theta }{\partial x_{i}}\right)}^{2}}}}.$$

Again, ${\bar{\Pi }}_{q}$ can be calculated via known mean quantities while $\Pi_{q}^{\prime }$ needs to be modeled. Considering the exact equation for thermal dissipation, $\varepsilon_{\theta }=2\alpha \bar{{\left(\partial \theta /\partial x_{i}\right)}^{2}}$, and thus by assuming a local equilibrium between $\varepsilon_{\theta }$ and term *B* entropy production due to heat transfer as well as using the Boussinesq approximation for the production term, discussed in Reference [45], Equation (24) can be approximated as follows:

(25) $$\Pi_{q}^{\prime }=\frac{\rho c_{p}}{T^{2}}\varepsilon_{\theta }.$$

Since $\varepsilon_{\theta }$ is not directly known without a transport equation, it can be calculated using the model given by Equation (14).

#### 2.4.2. Entropy Production—Unsteady Calculations

In contrast to the steady-state approach, the entropy production terms in Equation (17) must be split into res and sgs components as below:

(26) $$\Pi_{v}\approx \underset{\langle \Pi_{v}^{res}\rangle }{\underset{︸}{\langle \frac{\mu }{T}\left(\frac{\partial {\bar{u}}_{i}}{\partial x_{j}}+\frac{\partial {\bar{u}}_{j}}{\partial x_{i}}\right)\frac{\partial {\bar{u}}_{i}}{\partial x_{j}}\rangle }}+\underset{\langle \Pi_{v}^{sgs}\rangle }{\underset{︸}{\left(\langle \Pi_{v}\rangle -\langle \frac{\mu }{T}\left(\frac{\partial {\bar{u}}_{i}}{\partial x_{j}}+\frac{\partial {\bar{u}}_{j}}{\partial x_{i}}\right)\frac{\partial {\bar{u}}_{i}}{\partial x_{j}}\rangle \right)}},$$

(27) $$\Pi_{q}\approx \underset{\langle \Pi_{q}^{res}\rangle }{\underset{︸}{\langle \frac{\lambda }{T^{2}}\frac{\partial T}{\partial x_{i}}\;\frac{\partial T}{\partial x_{i}}\rangle }}+\underset{\langle \Pi_{q}^{sgs}\rangle }{\underset{︸}{\left(\langle \Pi_{q}\rangle -\langle \frac{\lambda }{T^{2}}\frac{\partial T}{\partial x_{i}}\;\frac{\partial T}{\partial x_{i}}\rangle \right)}},$$

where 〈()〉 donates spatial and time averaging (ensemble averaging). The res components can be calculated via known mean quantities while sgs components will be approximated following [7] as below:

(28) $$\langle \Pi_{v}^{sgs}\rangle \approx \frac{\rho }{T}\langle \varepsilon_{sgs}\rangle ,$$

(29) $$\langle \Pi_{q}^{sgs}\rangle \approx \frac{c_{p}\rho }{T^{2}}\langle {\varepsilon_{\theta }}_{sgs}\rangle .$$

## 3. Numerical Setup

The Reynolds Analogy is assessed using previously mentioned turbulence models at different Reynolds and Prandtl numbers. The details of the numerical schemes and the respective flow configuration are described in the following section.

### 3.1. Flow Configuration

**Figure 2:**
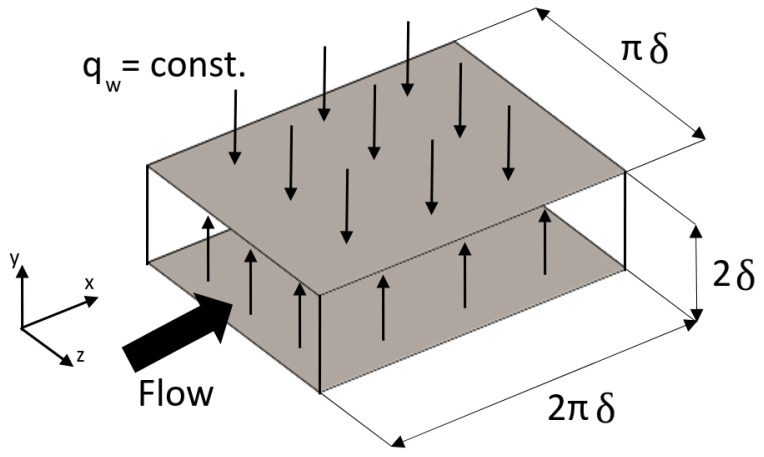
Sketch of horizontal channel flow configuration.

The configuration is a fully developed turbulent channel flow, shown in Figure 2. The size of the computational domain is 2πδ,2δ, and πδ. Different Reynolds and Prandtl numbers have been considered based on the availability of reference (DNS) data. The details of all simulations are summarized in Table 1. Note that the Reynolds number is defined based on the friction velocity at wall ($U_{\tau }$) and channel half height δ. A constant pressure gradient is applied via an additional source term in the momentum equation to drive the flow to the targeted Reynolds number. Periodic boundary conditions are imposed in the streamwise and the spanwise directions, and no-slip condition is used at both walls. For the temperature field, a mean uniform heat flux at the walls and periodic boundary conditions in the streamwise and the spanwise directions have been applied. Further, it is important to mention that the temperature variance is set to zero at the wall. Detailed information on the influence of the boundary condition can be found in References [37,40,47]. The results are normalized by the channel half width δ, the friction velocity $U_{\tau }$, the kinematic viscosity ν, the density ρ, the friction temperature $T_{\tau }$, and the friction entropy production rate $S_{\tau }$.

Detailed information on the mesh resolutions used for the unsteady-state simulations are given in Table 2. A simple gradient spacing is used to achieve appropriate distribution in the wall-normal direction. Further, it should be noted that the stretch factor **r** should be less than ≈1.2 [48,49], which is fulfilled for all meshes.

It is worth mentioning that, to the best of authors’ knowledge, no explicit DNS data are available on the entropy generation in a fully developed turbulent channel flow, i.e., entropy production has been calculated using available DNS data for quantities such as velocity and temperature as input data for the relations discussed in Section 2.4. For this study, the required DNS data have been taken from References [35,36].

### 3.2. Code Description

All numerical simulations presented in this work are performed using OpenFOAM-v1706 with necessary modifications for the purpose of this paper. PISO (Pressure-Implicit with Splitting of Operators) algorithm has been used for steady and unsteady calculations. Second-order schemes have been used for velocity, turbulence, and thermal quantities for both steady and unsteady simulations. Further, a Courant number around 0.05 was chosen for a reliable prediction of the velocity and temperature field for unsteady calculations as suggested in Reference [31].

## 4. Results and Discussion

In the framework of the present study, prediction capabilities of the Reynolds Analogy in accordance with the second law of thermodynamics for turbulent thermal effects at different Reynolds and Prandtl numbers are investigated, as provided in Table 1. This covers a wide range of Pr numbers, i.e., Pr=0.025,0.71, and 200, to study capabilities of the when dynamics of heat transfer are significantly different. The main goal here is to provide an assessment by investigating the entropy prediction capabilities of the. The results obtained from different simulations will be explained and discussed in the following.

### 4.1. Steady-State Simulations

The steady-state simulations are carried out using the k−ω−SST and ζ−f RANS-based models. It should be noted that mesh convergence studies have been done for all simulations. While only mesh independent results are presented, the detailed analyses can be found in Reference [50].

#### 4.1.1. Pr=0.71

**Figure 3:**
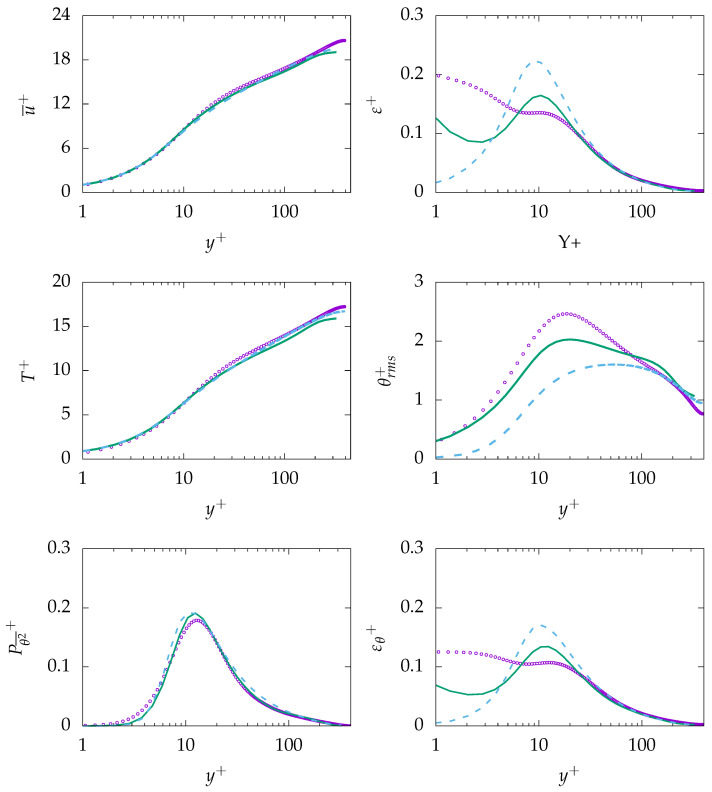
Evolution of streamwise velocity (**top left**), dissipation of *k* (**top right**), temperature (**middle left**), temperature root mean square (rms) (**middle right**), production of $\bar{\theta^{2}}$ (**bottom left**), and dissipation of $\bar{\theta^{2}}$ (**bottom right**) at ${Re}^{\tau }=395$ for Pr=0.71. ζ−f: 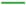, k−ω−SST: 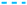, DNS: 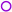.

Figure 3 presents mean velocity, dissipation of turbulent kinetic energy *k*, mean temperature, root mean square (rms) value of temperature fluctuations, as well as production and dissipation of $\bar{\theta^{2}}$ at ${Re}^{\tau }=395$ for Pr=0.71. As expected, mean velocity and mean temperature profiles are in good agreements with the DNS data. In contrast, ε is mispredicted in the near-wall region and shows only good agreement with DNS data after the buffer layer, i.e., $y^{+}>30$. Further, the rms value of temperature fluctuation ($\theta^{rms}$) is mispredicted by both turbulence models. A detailed analysis of the transport equation for $\bar{\theta^{2}}$ (Equation (13)) will help to understand the reason behind the misprediction. The production of $\bar{\theta^{2}}$ is well predicted, indicating that the production is primary due to the temperature gradient in the wall-normal direction. However, the thermal dissipation $\varepsilon_{\theta }$ is mispredicted particularly in the near-wall region, which is thought to be the main reason of misprediction of $\bar{\theta^{2}}$ and, thus, $\theta^{rms}$. For this study, the assumption of a constant thermal to mechanical time-scale ratio (R) is used to derive $\varepsilon_{\theta }$. This assumption describes $\varepsilon_{\theta }$ based on ε, which could lead to misprediction of $\varepsilon_{\theta }$ in the near-wall region, since ε is mispredicted in the near-wall region.

**Figure 4:**
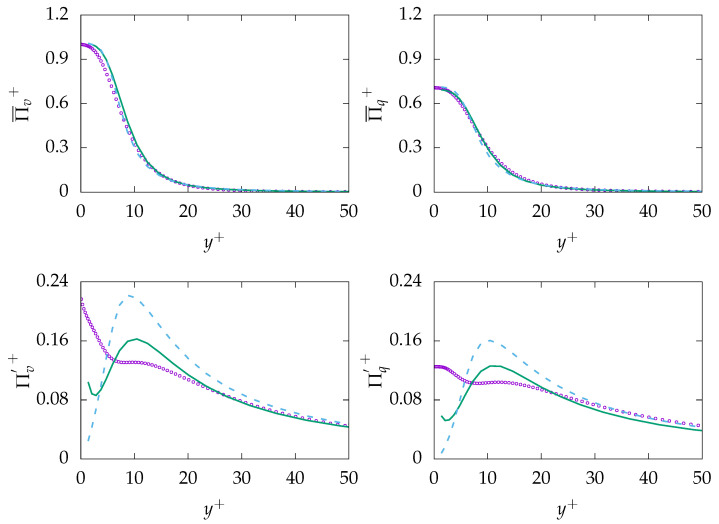
Evolution of mean entropy production (**top**) $\overset{¯}{\Pi_{i}}$ and fluctuation entropy production $\Pi_{\prime }^{i}$ (**bottom**) due to viscous dissipation (*v*, **left**) and heat transfer (*q*, **right**) at ${Re}^{\tau }=395$ for Pr=0.71. ζ−f: 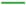, k−ω−SST: 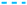, DNS: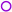.

Figure 4 presents entropy production due to viscous dissipation (mean and fluctuation) and production due to heat transfer (mean and fluctuation). It can be observed that both mean entropy generations are well predicted as they are directly related to the mean velocity and temperature, which are well predicted by both turbulence models. However, the entropy productions due to fluctuations are mispredicted in the near-wall region for both generation mechanisms. Further away from the wall, both fluctuation quantities follow a very similar tendency compared to DNS data. Moreover, the assumption of a constant thermal to mechanical ratio (constant R) seems to be reasonable for fluids with Pr number around unity, as ε and $\varepsilon_{\theta }$ show pretty much similar dynamics as shown in Figure 3. However, more advanced models for ε are required to accurately predict ε and, consequently, $\varepsilon_{\theta }$ as well as entropy generation by fluctuations in the near-wall region. Furthermore, it can be seen that both entropy generation mechanisms almost equally contribute to the total amount of entropy generated in the process.

#### 4.1.2. Pr=0.025

**Figure 5:**
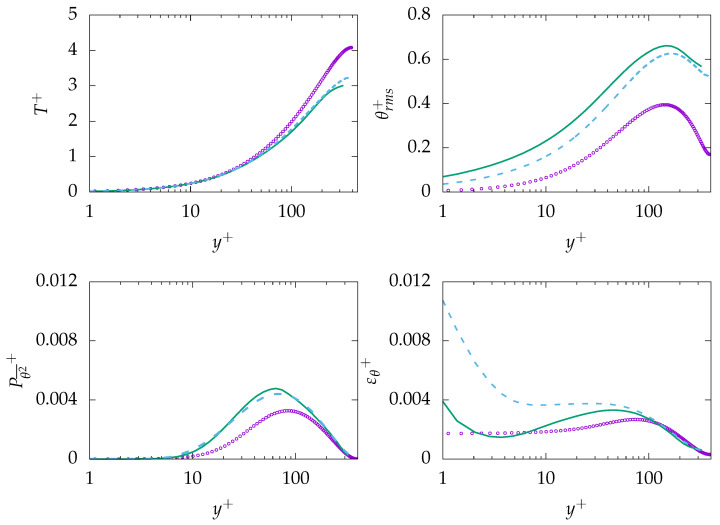
Evolution of temperature (**top left**), temperature rms (**top right**), production of $\bar{\theta^{2}}$ (**bottom left**), and dissipation of $\bar{\theta^{2}}$ (**bottom right**) at ${Re}^{\tau }=395$ for Pr=0.025. ζ−f: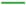, k−ω−SST: 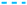, DNS: 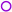.

The simulation for Pr=0.025 is carried out at ${Re}^{\tau }=395$, as provided in Table 1. It should be noted that the flow field results are not shown as the temperature is considered to be a passive scalar. Figure 5 presents mean temperature and temperature rms profiles along with production and dissipation of $\bar{\theta^{2}}$. In contrast to the previous simulation concerning Pr=0.71, there are discrepancies in mean temperature (underprediction of ≈25%) and temperature variance (equivalently $\theta^{rms}$) is severely overpredicted over the whole channel domain. As discussed in Reference [50] and shown in Figure 1, the misprediction of the temperature is likely a result of the assumption of a constant turbulent Prandtl number in the Reynolds Analogy. Further, the overprediction of the production of $\bar{\theta^{2}}$ leads to the discrepancy in $\theta^{rms}$. However, the situation is worse for the dissipation: as shown, both turbulence models fail to predict the plateau behavior of $\varepsilon_{\theta }$. Furthermore, it clearly can be seen that the assumption of constant thermal to mechanical time scale (R) is not reasonable for fluids with numbers significantly less than unity, as ε (shown in Figure 3) indicates completely different tendency compared to $\varepsilon_{\theta }$—in contrast to fluids with Pr number around unity.

**Figure 6:**
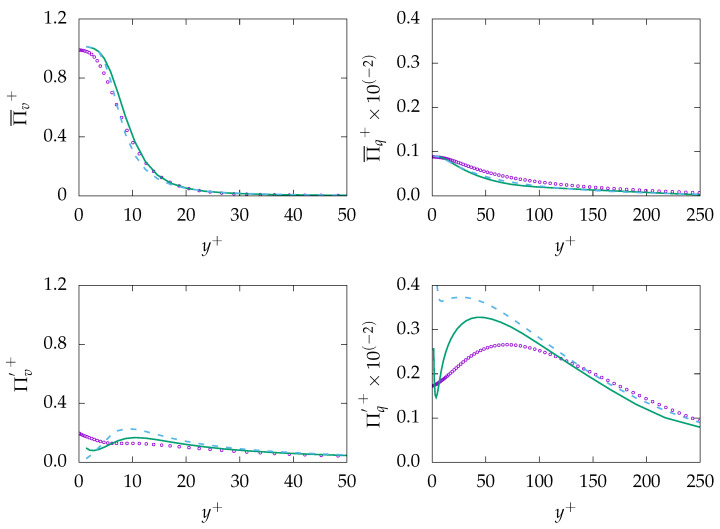
Evolution of mean entropy production $\overset{¯}{\Pi_{i}}$ (**top**) and fluctuation entropy production $\Pi_{\prime }^{i}$ (**bottom**) due to viscous dissipation (*v*, **left**) and heat transfer (*q*, **right**) at ${Re}^{\tau }=395$ for Pr=0.025. ζ−f: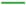, k−ω−SST: 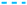, DNS: 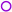.

Figure 6 presents entropy production due to viscous dissipation (mean and fluctuation) and production due to heat transfer (mean and fluctuation). As expected, mean entropy generation due to viscous dissipation is in reasonable agreement with the DNS data. Further, mean entropy generation due to heat transfer follows closely DNS data, with a slight deviation. Similar to the previous simulation, entropy generation due to the fluctuations is in the near-wall region not accurately predicted, mainly due to misprediction of ε and, accordingly, misprediction of $\varepsilon_{\theta }$ in near-wall region. However, the prediction is in good agreement with the reference data further away form the wall. It is worth mentioning that, in contrast to Pr=0.71, the total entropy generation and, therefore, the irreversibilities of the process mainly stem from the viscous dissipation as it dominates over the entropy production due to heat transfer. This is probably due to high thermal conductivity of fluids with low Pr numbers that allows an efficient heat transfer.

#### 4.1.3. Pr=200

In contrast to previous simulations, simulations for Pr=200 are carried out at ${Re}^{\tau }=150$ due lack of sufficient reference data at higher ${Re}^{\tau }$. It was shown in Reference [39] that, for Pr=0.71 and larger, temperature field data is roughly independent of ${Re}^{\tau }$ and the temperature field mainly depends on Pr number. It is worth mentioning that high Prandtl number fluids impose some computational challenges, and thus, certain mesh requirements need to be considered [36,50,51]. However, only mesh-independent results are presented in this study.

**Figure 7:**
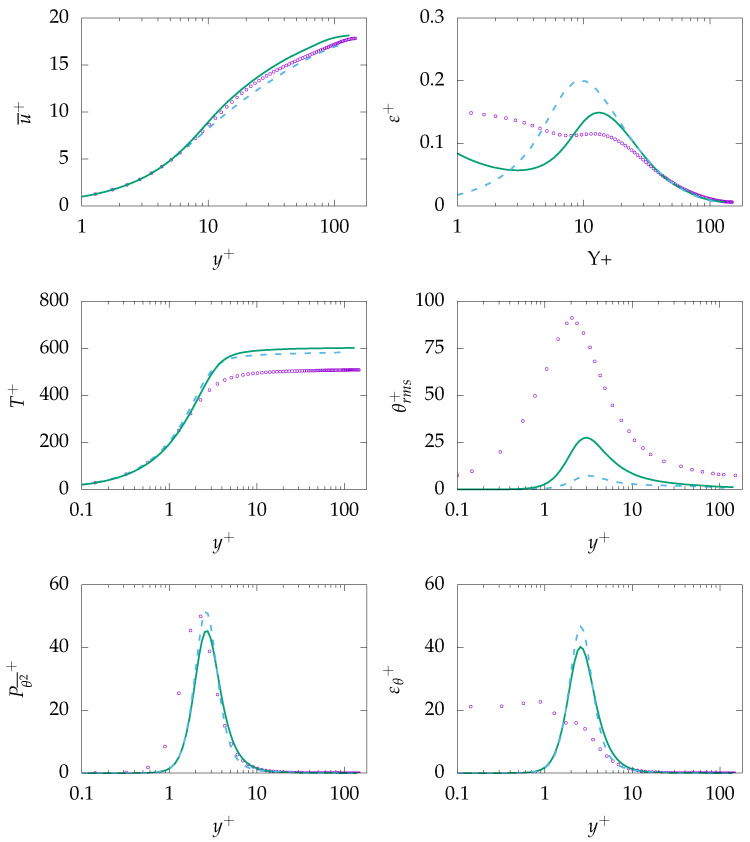
Evolution of streamwise velocity (**top left**), dissipation of *k* (**top right**), temperature (**middle left**), temperature rms (**middle right**), production of $\bar{\theta^{2}}$ (**bottom left**), and dissipation of $\bar{\theta^{2}}$ (**bottom right**) at ${Re}^{\tau }=150$ for Pr=200. ζ−f: 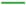, k−ω−SST: 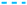, DNS: 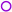.

Figure 7 presents mean velocity, dissipation of *k*, mean temperature, rms of temperature fluctuations, as well as production and dissipation of $\bar{\theta^{2}}$. It should be noted that, generally, turbulence models have been developed based on high Reynolds number assumption. Therefore, prediction quality of these models when dealing with relatively low Reynolds number, as in the present case, might be decreased [50], such as for the mean velocity profile, which is thought to be the main reason of the overprediction of mean temperature. In contrast to the mean temperature, $\theta^{rms}$ is strongly underpredicted by both turbulence models, mainly due to the misprediction of dissipation of $\bar{\theta^{2}}$, i.e., $\varepsilon_{\theta }$. While the production is in good agreement with DNS data, the dissipation in the near-wall region is severely mispredicted inside the thermal boundary layer, i.e., $y^{+}\approx 4$.

**Figure 8:**
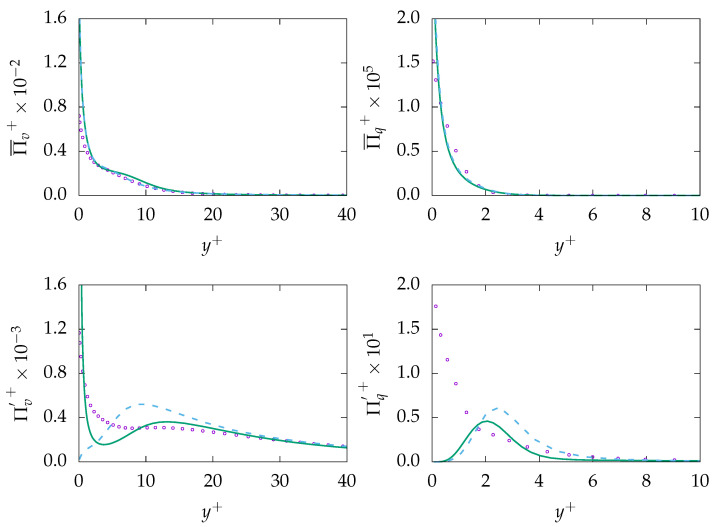
Evolution of mean entropy production $\overset{¯}{\Pi_{i}}$ (**top**) and fluctuation entropy production $\Pi_{\prime }^{i}$ (**bottom**) due to viscous dissipation (*v*, **left**) and heat transfer (*q*, **right**) at ${Re}^{\tau }=150$ for Pr=200. ζ−f: 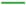, k−ω−SST: 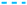, DNS: 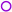.

Figure 8 presents entropy production due to viscous dissipation (mean and fluctuation) and production due to heat transfer (mean and fluctuation). It is worth mentioning that the very thin thermal boundary layer with its high temperature gradient is clearly visible, especially in the evaluation of $\Pi_{q}$, which vanishes for $y^{+}>3$. Again, it can be observed that both mean entropy generations, i.e., ${\bar{\Pi }}_{v}$ and ${\bar{\Pi }}_{q}$, are overall fairly well predicted as they are directly related to the mean velocity and temperature values, which are in good agreement with the DNS data for both turbulence models. Similarly, entropy production due to fluctuating quantities indicate acceptable predictions except for regions very close to the wall. More importantly, entropy generation due to heat transfer is the dominant mechanism, in contrast to previous simulation concerning low fluids. This is most likely due to very low thermal conductivity of the fluids, which leads to a very high temperature gradient at the surface to reach the targeted energy that needs to be transferred to the fluid at the wall via conduction.

### 4.2. Unsteady Simulations

Unsteady simulations have been carried out using the k−ω−SST-IDDES model. Three different Pr numbers, i.e., 0.71, 0.025, and 200, have been considered. All three Pr numbers are investigated with two different resolutions to demonstrate the influence of mesh resolution and, more importantly, to study the behavior of the when operating as an sgs model. Further, only results on adequate grids will be presented; for details, see Reference [50]. All presented results are spatial and time averaged, which corresponds to 〈()〉, as described in the nomenclature.

The k−ω−SST-IDDES model is a hybrid URANS/LES approach and is able to provide an accurate prediction of the flow field comparable to wall-modeled LES at affordable computational cost [28]. Furthermore, this model treats the near-wall region in the URANS-model, while transitioning to LES-mode away from the wall. This will allow investigation on the dynamics of the transition of the Reynolds Analogy from URANS to LES-mode, where this analogy operates as an sgs model.

#### 4.2.1. Pr=0.71

**Figure 9:**
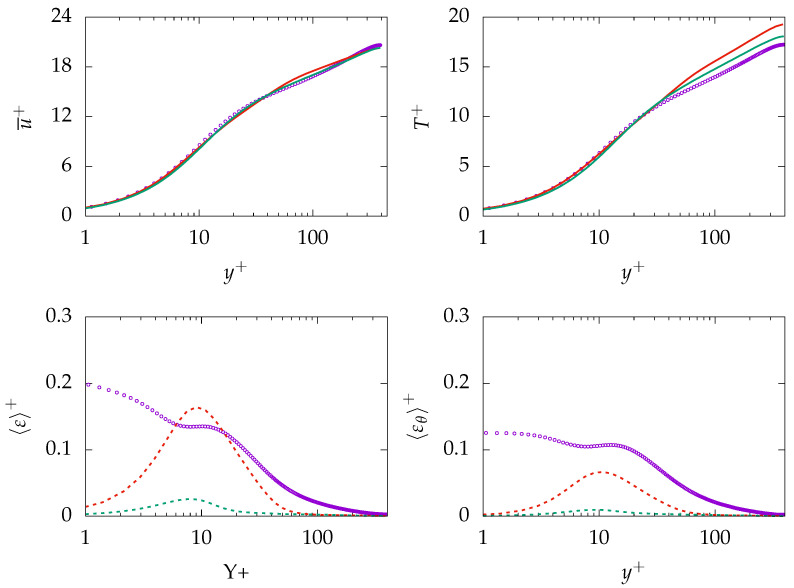
Evolution of the streamwise mean velocity (**top left**), mean temperature (**top right**), modeled dissipation of *k* (**bottom left**), and dissipation of $\bar{\theta^{2}}$ (**bottom right**) at ${Re}^{\tau }=395$ for Pr=0.71 obtained on different meshes. A-100: 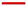, B-100: 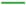, A-100: 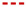, B-100: 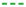, DNS: 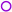.

Figure 9 presents the results obtained at ${Re}^{\tau }=395$ for Pr=0.71 on mesh A-100 and B-100. This includes mean velocity, mean temperature, and modeled viscous and thermal dissipations (ε and $\varepsilon_{\theta }$). It is important to mention that the resolved—and thus, total—components of ε and $\varepsilon_{\theta }$ are not presented because they are not contributing in the calculation of entropy generation.

It can be observed that the mean velocity is marginally influenced by mesh resolution. In contrast, the mean temperature improves with increasing the resolution. Further, the model is not capable of predicting the near-wall behavior of modeled ε and, consequently, $\varepsilon_{\theta }$. More importantly, both quantities vanish with increasing mesh resolution. This is particularly important for calculation of entropy production, as the modeled part of ε and $\varepsilon_{\theta }$ contribute to determining irreversibilities of the process. However, the operating as an sgs model for thermal effects within IDDES methodology indicates similar response to mesh resolution as the flow quantities, i.e., *k* and ε. This has been discussed in detail in Reference [50]. Vanishing of modeled ε and $\varepsilon_{\theta }$ in response to mesh refinement cannot be considered appropriate, as the fine resolution is still too coarse to support DNS. Therefore, the needs to be cautiously applied in unsteady simulations as it may fail to capture phenomena that mostly occur at the small scale/sgs level.

**Figure 10:**
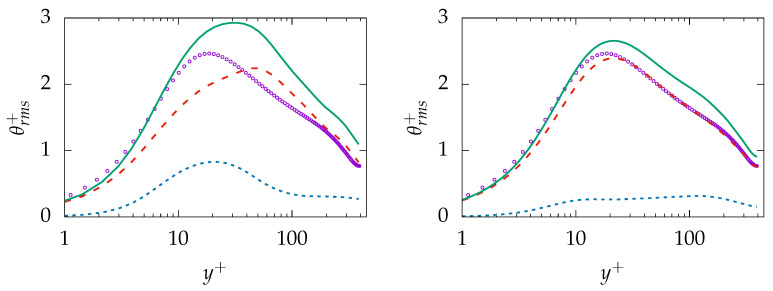
Evolution of $\theta^{rms}$ at ${Re}^{\tau }=395$ for Pr=0.71 obtained on different meshes; A-100 (**left**) and B-100 (**right**). total: 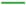, resolved: 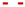, modeled: 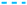, DNS: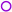.

Similar behavior is present in the prediction of $\theta^{rms}$; see Figure 10. The near-wall behavior of the total quantity is in acceptable agreement with DNS data while the behavior further away is mispredicted on the coarse mesh (A-100). More importantly, the results are improved on the finer resolution (B-100) and the resolved part of $\theta^{rms}$ is well predicted while the sgs part shows rather a nonphysical plateau profile. Thus, it may be concluded that the model tries to resolve most of thermal structures irrespective of mesh resolution.

**Figure 11:**
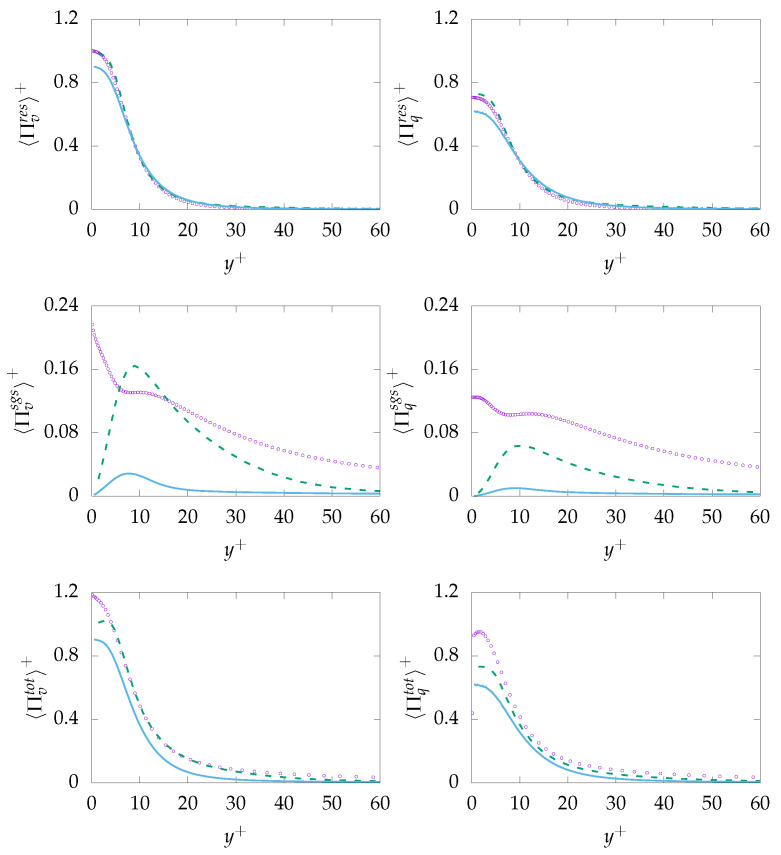
Evolution of entropy production due to resolved $\langle \Pi_{res}^{q}\rangle$ (**top**), sub-grid $\langle \Pi_{sgs}^{i}\rangle$ (**middle**) and total $\langle \Pi_{tot}^{i}\rangle$ (**bottom**) parts due to viscous dissipation (*v*, **left**) and heat transfer (*q*, **right**) at ${Re}^{\tau }=395$ for Pr=0.7 obtained on different meshes. A-100: 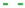, B-100: 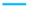, DNS: 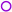.

The entropy production obtained on both mesh resolutions is given in Figure 11. As expected, both resolved quantities, i.e., $\langle \Pi_{res}^{v}\rangle$ and $\langle \Pi_{res}^{q}\rangle$, are well predicted with a negligible discrepancy at the wall. However, the modeled/sgs parts are not predicted accurately due to inaccurate prediction of ε and, consequently, $\varepsilon_{\theta }$. It is worth mentioning that the reduction of the modeled part is a consistent response to mesh refinement. However, the extend of the reduction (vanishing) on a mesh that cannot support DNS is concerning. Regarding total entropy production due to both mechanisms, it can be observed that results obtained on the coarse mesh are in better agreement with the DNS data compared to results obtained on the fine mesh. This will lead to the conclusion that the k−ω−SST-IDDES model tries to resolve most structures especially on the fine mesh but fails to improve the resolved quantities accordingly.

#### 4.2.2. Pr=0.025

It is shown in Reference [50] that mesh design plays an integral role in capturing thermal statistics at low Pr numbers in unsteady-state simulations, and thus, only appropriate grids are employed for this study; see Table 2 for details. It was shown that mesh needs to be close to isotropic in the core region of channel in order to accurately resolve thermal structures. Furthermore, it should be noted that the temperature is a passive scalar, and thus, the flow quantities are not presented again.

**Figure 12:**
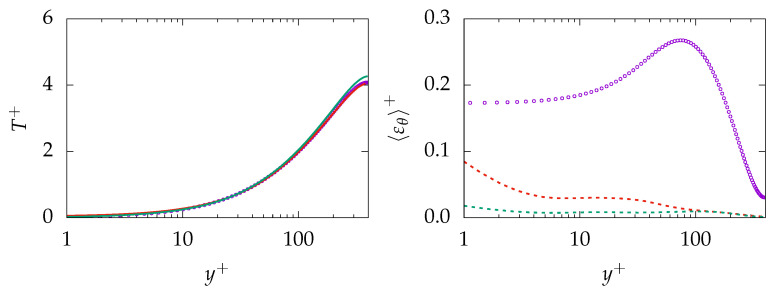
Evolution of the mean temperature (**left**) and modeled dissipation of $\varepsilon_{\theta }$ (**right**) at ${Re}^{\tau }=395$ for Pr=0.025 obtained on different meshes. A-100: 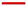, B-100: 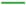, A-100: 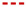, B-100: 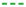, DNS: 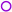.

**Figure 13:**
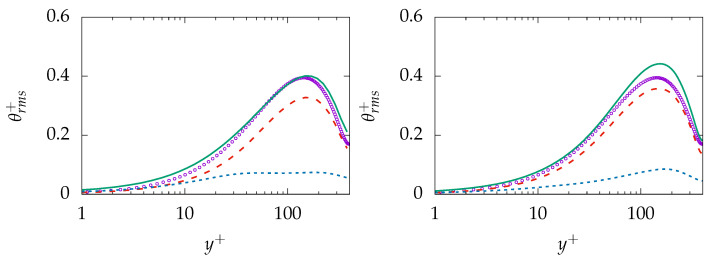
Evolution of $\theta^{rms}$ at ${Re}^{\tau }=395$ for Pr=0.025 obtained on different meshes; A-100 (**left**) and B-100 (**right**). total: 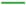, resolved: 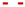, modeled: 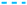, DNS: 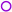.

Results obtained for mean temperature and modeled $\varepsilon_{\theta }$ on mesh A-100 and B-100 are presented in Figure 12. The temperature profile is well predicted on both grids and shows no remarkable sensitivity regarding the mesh resolution. As expected, modeled $\varepsilon_{\theta }$ is mispredicted on both grids over the whole domain and vanishes with increasing resolution. In contrast, the prediction of $\theta^{rms}$ shows a slight mesh sensitivity; see Figure 13. The IDDES model tries to resolve $\theta^{rms}$ completely and pushes the simulation towards DNS. However, the modeled part does not vanish completely and, finally, leads to a slight overprediction on the fine mesh.

**Figure 14:**
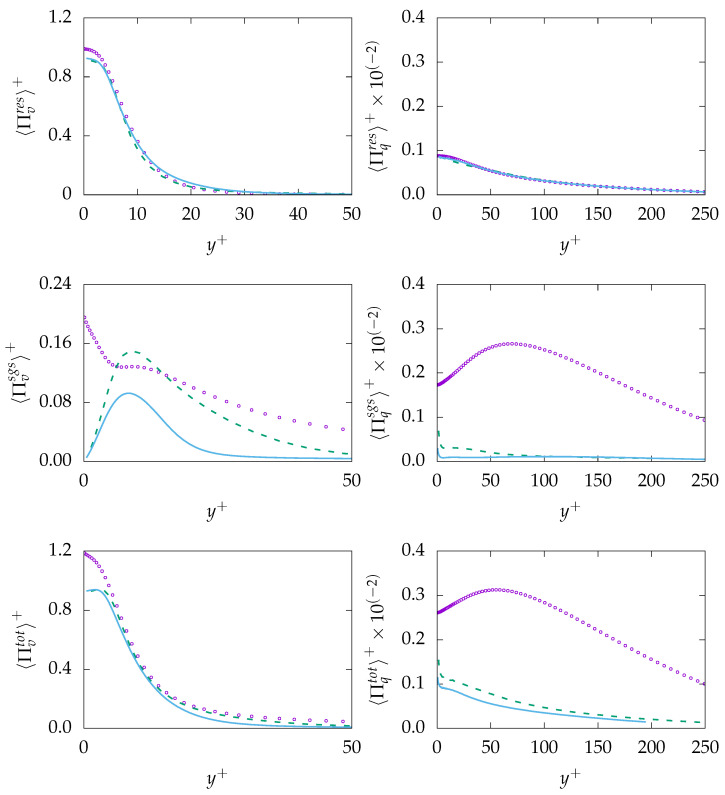
Evolution of entropy production due to resolved $\langle \Pi_{res}^{i}\rangle$ (**top**), sub-grid $\langle \Pi_{sgs}^{i}\rangle$ (**middle**) and total $\langle \Pi_{tot}^{i}\rangle$ (**bottom**) parts due to viscous dissipation (*v*, **left**) and heat transfer (*q*, **right**) at ${Re}^{\tau }=395$ for Pr=0.025 obtained on different meshes. A-100: 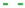, B-100: 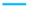, DNS: 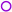

The entropy production obtained on both mesh resolutions is given in Figure 14. As expected, both resolved quantities, i.e., $\langle \Pi_{res}^{v}\rangle$ and $\langle \Pi_{res}^{q}\rangle$ show reasonable agreement with the DNS data. However, the sgs-entropy generation, i.e., $\langle \Pi_{res}^{v}\rangle$ and $\langle \Pi_{res}^{q}\rangle$, are severely mispredicted. This is mainly due to the fact that the viscous dissipation rate ε is not accurately predicted and that, consequently, the thermal dissipation rate $\varepsilon_{\theta }$ suffers from the same misprediction. Total entropy production due to viscous dissipation $\langle \Pi_{tot}^{v}\rangle$ is in good agreement with DNS data. Similar to previous simulation for Pr=0.71, the prediction capabilities decrease slightly with increasing resolution. However, $\langle \Pi_{tot}^{q}\rangle$ is mispredicted over the whole domain. Taking into account that, in contrast to Pr=0.71, the sgs part of entropy production due to heat transfer is roughly twice as big than the res part, the incapability of the methodology to predict the sgs part accurately is believed to be the reason for the misprediction of $\langle \Pi_{tot}^{q}\rangle$.

The results suggest that the main assumption of the —strong similarity between mechanical and thermal fields in combination with a constant thermomechanical time scale R—is facing severe challenges in case of fluids with Pr numbers significantly less than unity, calling for more advanced models for the heat flux as well as for $\varepsilon_{\theta }$.

Similar to the previous RANS simulations concerning =0.025, comparing total entropy production due to the viscous dissipation and heat transfer leads to the conclusion that viscous dissipation is the dominant mechanism, causing most irreversibilities of processes dealing with low Pr fluids.

#### 4.2.3. Pr=200

Simulations for Pr=200 have been performed at ${Re}^{\tau }=150$ on two different resolutions; see Table 1. As mentioned before, capturing the thermal effects at high Pr numbers fluids is very challenging as the thermal boundary layer is very thin—compared to the boundary of the flow, which leads to very dominant wall effects. As a result, investigating thermal boundary layers at high Pr numbers are limited to relatively low ${Re}^{\tau }$ due to the prohibitively expensive computational cost [36,51].

**Figure 15:**
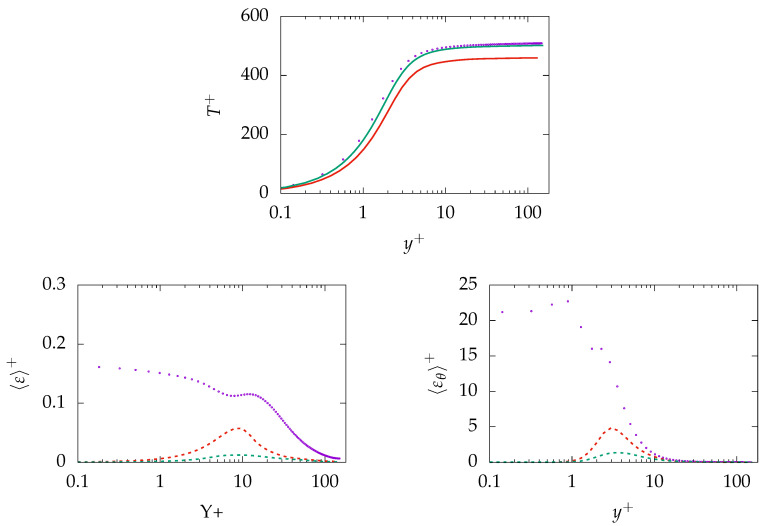
Evolution of mean temperature (**top**), modeled dissipation of *k* (**bottom left**), and dissipation of $\bar{\theta^{2}}$ (**bottom right**) at ${Re}^{\tau }=150$ for Pr=200 obtained on different meshes. A-1000: 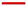, C-250: 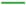, A-1000: 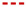, C-250: 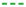, DNS: 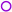.

Results obtained for mean temperature, modeled dissipation of *k*, and $\bar{\theta^{2}}$ on both meshes are shown in Figure 15. Concerning temperature profile, the result is underpredicted on the coarse grid (A-1000) by roughly 10%. However, the prediction improves on the fine mesh (B-250) and the profile is in good agreement with DNS data. Regarding modeled dissipation of *k*, the quantity is mispredicted especially close to the wall. Furthermore, the situation is worse for modeled $\varepsilon_{\theta }$ where the near-wall region is completely mispredicted.

**Figure 16:**
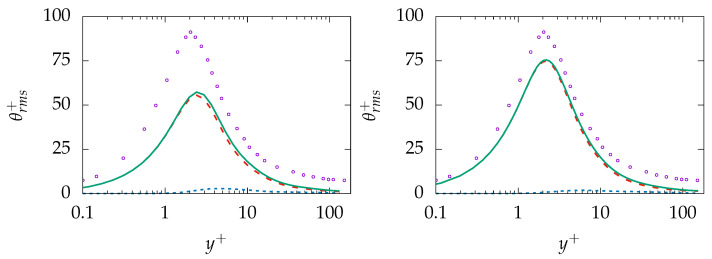
Evolution of $\theta^{rms}$ at ${Re}^{\tau }=150$ for Pr=200 obtained on different meshes; A-1000 (**left**) and C-250 (**right**)). total: 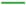, resolved: 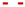, modeled: 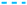, DNS: 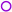.

$\theta^{rms}$ obtained on the same grids is presented in Figure 16. The profile is underpredicted over the whole domain with the negligible modeled part on the coarse mesh. The sgs model is incapable of capturing the near-wall dynamics and provides appropriate results, while the resolution is too coarse to capture dynamics of $\theta^{rms}$. While general improvement can be observed for $\theta^{rms}$ on the finer mesh, the mesh resolution is not fine enough to deliver acceptable results for the thermal second-order statistics.

**Figure 17:**
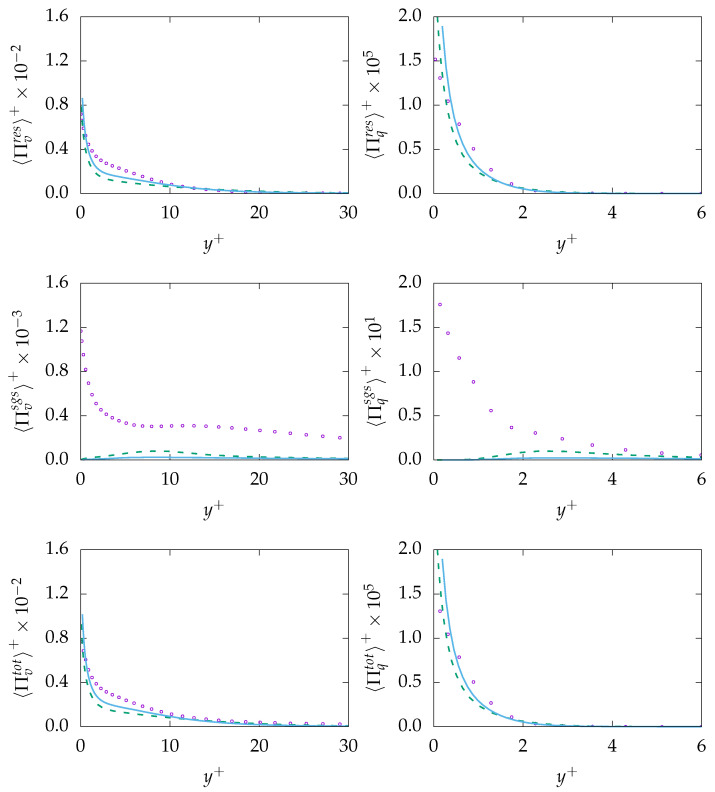
Evolution of entropy production due to resolved $\langle \Pi_{res}^{q}\rangle$ (**top**), sub-grid $\langle \Pi_{sgs}^{i}\rangle$ (**middle**) and total $\langle \Pi_{tot}^{i}\rangle$ (**bottom**) parts due to viscous dissipation (*v*, **left**) and heat transfer (*q*, **right**) at ${Re}^{\tau }=150$ for Pr=200 obtained on different meshes. A-1000: 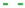, C-250: 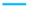, DNS: 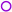.

Figure 17 demonstrates the entropy generation due to different mechanism for Pr=200. As expected, the entropy generation due to resolved quantities, i.e., $\langle \Pi_{res}^{v}\rangle$ and $\langle \Pi_{res}^{q}\rangle$, are well predicted with no significant sensitivity to grid resolution. In contrast, the entropy generation due to the sgs model, e.g., $\langle \Pi_{res}^{v}\rangle$ and $\langle \Pi_{res}^{q}\rangle$, is mispredicted mainly due to the misprediction of the modeled dissipation rate ε and, consequently, the modeled thermal dissipation rate $\varepsilon_{\theta }$. However, in contrast to previous cases, sgs parts of entropy generation play a minor role compared to the res part. Thus, the misprediction of sgs parts is not notably present in the total value.

The results obtained for Pr=200 suggest that the is not playing an integral part to model sub-grid thermal effects. This analogy fails to feature basic property of an appropriate sgs model in a mesh with a coarser resolution than DNS, i.e., there is basically no modeled part for temperature variance and entropy. Therefore, application of this analogy to capture near-wall thermal phenomena in complex high Prandtl number flows where providing high enough resolution is not feasible might lead to significant inaccuracies.

However, despite the issue discussed, the total entropy generation obtained from both mechanisms confirm the finding of RANS simulation that most of the irreversibility of processes dealing with high Pr number fluids stem from thermal phenomena.

## 5. Conclusions and Outlook

In this study, predictive capabilities of the to determine entropy production mainly through heat transfer has been thoroughly assessed. This includes application of this analogy to turbulent wall-bounded shear flows at different Reynolds and Prandtl numbers within steady- and unsteady-state calculations. In case of steady-state calculations, the is able to provide acceptable results for mean and fluctuating entropy generation for Prandtl numbers around unity. Departing away from these Prandtl numbers, the is still capable of predicting the mean entropy production in good agreement with DNS data. However, the fluctuating production fails particularly in the near-wall region, mainly due to the misprediction of the dissipation of kinetic energy.

Concerning unsteady calculations, it was shown that the fails to feature basic properties of an appropriate sub-grid scale model, mainly due to inappropriate response to mesh resolution. Further, the mean entropy generation is well predicted for all investigated Prandtl numbers. Concerning the sub-grid model properties, the model pushes the simulations towards direct numerical simulation on any grid resolution, leading to misprediction of sub-grid values such as sub-grid entropy production particularly for low and high Prandtl numbers. This could lead to significant error when near-wall phenomena and/or fluctuations are of great importance, and thus, the may not be considered as a reliable sub-grid scale modeling strategy. Moreover, results suggest that optimization efforts need to be put on minimizing viscous dissipation for processes involving low Prandtl number fluids while efficient heat transfer is the key to reducing irreversibility of a process dealing with high Prandtl number fluids. Further, it turns out that both mechanisms for entropy generation are equally important concerning fluids with Prandtl number around unity, suggesting necessity of concurrent optimization to reduce viscous dissipation while making heat transfer more efficient. This clearly makes optimization a more challenging task.

The obtained results confirm that using the zero-equation approach (the) cannot be deemed as an appropriate tool for design and optimization purposes, especially when relying on entropy generation/optimization strategies and working fluids with non-unity Prandtl numbers. This strongly suggests moving toward development of more advanced turbulent heat transfer models consistent with thermodynamics laws, which requires application of one-equation or algebraic models to model heat transfer phenomena [43] in conjunction with advanced turbulence models capable of capturing complex and nonlinear wall effects.

## Figures and Tables

**Table 1:** Overview of simulations.

Reτ	Pr	Reference	Type	Resolution	Grids
395	0.025	Kawamura et al. [35]	steady	48×72×48	A-100
			unsteady		
			unsteady	128×192×48	B-100
395	0.71	Kawamura et al. [35]	steady	48×72×48	A-100
			unsteady		
			unsteady	128×192×48	B-100
150	200	Bergant et al. [36]	steady	48×72×48	A-1000
			unsteady		
			unsteady	128×192×96	C-250

**Table 2:** Details of the grid resolution for fully developed turbulent channel flow for unsteady-state simulations.

Reτ	Grids	$\Delta x^{+}$	$\Delta y_{w}^{+}-\Delta y_{c}^{+}$	$\Delta z^{+}$	$N_{x}$	$N_{y}$	$N_{z}$	r
395	DNS [35]	9.88	0.15–6.52	4.59	512	192	512	-
	Mesh A-100	51.7	0.49–19.1	25.9	48	72	48	1.14
	Mesh B-100	19.5	0.19–19.0		128	192		1.05
150	DNS [36]	12.3	0.04–3.3	4.6	192	145	128	-
	Mesh A-1000	19.5	0.03–27.7	9.7	48	72	48	1.21
	Mesh C-250	7.5	0.03–8.7	5.1	128	192	96	1.06

## Nomenclature

$c_{p}$	specific heat capacity at constant pressure
*f*	elliptic relaxation
*k*	kinetic energy
r	grid stretching factor in y-direction
*s*	entropy density
$u_{i}$	velocity fluctuations
${\bar{u}}_{i}$	mean velocity
$q_{i}$	heat flux density vector
$q_{w}$	wall heat flux
$y^{+}$	yPlus
Pr	Prandtl
$R=\tau_{\theta }/\tau_{m}$	mechanical to thermal time-scale ratio
${Re}^{\tau }=U_{\tau }\delta /\nu$	turbulent Reynolds number
$S_{\tau }=\nu \alpha {\left(T_{w}/T_{\tau }\right)}^{2}/U_{\tau }\lambda$	friction entropy production rate
$S_{T}$	source terms in internal temperature equation
*T*	mean temperature
$T_{\tau }=\rho /c_{p}q_{w}U_{\tau }$	friction temperature
$T_{w}$	wall temperature
$U_{\tau }={Re}^{\tau }\nu /\delta =\sqrt{\tau_{w}/\delta }$	friction velocity
$U_{i}={\bar{u}}_{i}+u_{i}$	total velocity
$\alpha =\lambda /\rho c_{p}$	thermal diffusivity
δ	channel half height
ϵ	dissipation of kinetic energy
$\varepsilon_{\theta }$	dissipation of the variance of temperature fluctuations
$\zeta =\bar{v^{2}}/k$	velocity scale ratio
θ	temperature fluctuations
$\bar{\theta u_{i}}$	turbulent heat flux
$\theta^{rms}$	rms value of temperature fluctuations
$\bar{\theta^{2}}$	temperature variance
Θ=T+θ	total temperature
ω	dissipation rate of kinetic energy
λ	thermal conductivity
μ	dynamic viscosity
ν	kinematic viscosity
$\nu_{t}$	turbulent kinematic viscosity
Π	entropy production
ρ	density
$\sigma_{t}$	turbulent Prandtl number
$\tau_{m}=k/\varepsilon$	mechanical time scale
$\tau_{\theta }=\bar{\theta^{2}}/2\varepsilon_{\theta }$	thermal time scale
$\tau_{w}$	wall shear stress
${\left(\;\right)}^{sgs}$	sub-grid component
${\left(\;\right)}^{res}$	resolved component
${\left(\;\right)}^{tot}$	total
$\bar{\left(\;\right)}$	mean value
〈()〉	spatial and time averaged
${\left(\;\right)}^{\prime }$	fluctuating component
${\left(\;\right)}^{+}$	normalized by wall variables

## Abbreviations

The following abbreviations are used in this manuscript:

res	resolved
rms	root mean square
sgs	sub-grid-scale
AFM	algebraic heat flux model
CFD	Computational Fluid Dynamics
DNS	Direct Numerical Simulation
EDM	Eddy Diffusivity model
GGDH	generalized gradient diffusion hypothesis
IDDES	improved delayed ettached Eddy simulation
SGDH	Simple Gradient Diffusion Hypothesis
LES	Large Eddy Simulation
RANS	Reynolds-Averaged Navier Stokes equation
URANS	Unsteady Reynolds-Averaged Navier Stokes equation
THF	turbulent heat flux
